# The Correlation between Cardiac Magnetic Resonance Findings and Post-COVID-19: The Impact of Myocardial Injury on Quality of Life

**DOI:** 10.3390/diagnostics14171937

**Published:** 2024-09-02

**Authors:** Maja Popovic, Vladimir Cvetic, Viseslav Popadic, Kristina Ilic, Aleksandra Radojevic, Andrea Klasnja, Natasa Milic, Nina Rajovic, Ratko Lasica, Drasko Gostiljac, Slobodan Klasnja, Edvin Mahmutovic, Marija Zdravkovic

**Affiliations:** 1 Department for Radiology, University Hospital Medical Center Bežanijska kosa, 11000 Belgrade, Serbia; 2 Department for Cardiovascular Radiology, University Clinical Centre of Serbia, 11000 Belgrade, Serbia; 3 Faculty of Medicine, University of Belgrade, 11000 Belgrade, Serbia; 4 Department for Cardiology, University Hospital Medical Center Bežanijska kosa, 11000 Belgrade, Serbia; viseslavpopadic@gmail.com (V.P.);; 5 Institute for Medical Statistics and Informatics, Faculty of Medicine, University of Belgrade, 11000 Belgrade, Serbia; 6 Department of Internal Medicine, Division of Nephrology and Hypertension, Mayo Clinic, Rochester, MN 55905, USA; 7 Clinic of Cardiology, Clinical Center of Serbia, 11000 Belgrade, Serbia; 8 Clinic of Endocrinology, Diabetes and Metabolic Diseases, Clinical Center of Serbia, 11000 Belgrade, Serbia; 9 General Hospital of Novi Pazar, 36300 Novi Pazar, Serbia

**Keywords:** COVID-19, cardiovascular symptoms, cardiac magnetic resonance

## Abstract

Background: In the post-COVID-19 era, there is growing concern regarding its impact on cardiovascular health and the following effects on the overall quality of life of affected individuals. This research seeks to investigate cardiac magnetic resonance (CMR) findings following COVID-19 and their impact on the quality of life of affected individuals. Methods: An observational, cross-sectional study was conducted in consecutive patients with persistent cardiovascular symptoms after COVID-19 who were referred to CMR due to suspected myocardial injury. In addition, patients completed a questionnaire about symptoms and the quality of life during the post-COVID-19 period. Results: In this study, 85 patients were included. The study population consisted of patients with a mean age of 42.5 ± 13.4 years, predominantly women, who made up 69.4% of the study population, while men made up 30.6%. CMR findings showed non-ischemic myocardial injury in 78.8% of patients and myocardial edema in 14.1% of patients. Late pericardial enhancement was present in 40% of patients and pericardial effusion in 51.8% of patients. Pericardial effusion (*p* = 0.001) was more prevalent in patients who reported more pronounced symptoms in the post-COVID-19 period compared to the acute infection phase. Predictors of lower quality of life in the post-COVID-19 period were the presence of irregular heartbeat (*p* = 0.039), cardiovascular problems that last longer than 12 weeks (*p* = 0.018), and the presence of pericardial effusion (*p* = 0.037). Conclusion: Acute myocarditis was observed in a minority of patients after COVID-19, while non-ischemic LGE pattern and pericardial effusion were observed in the majority. Quality of life was worse during the post-COVID-19 period in patients with CMR abnormalities, primarily in patients with pericardial effusion. Also, irregular heartbeat, cardiovascular symptoms that last longer than 12 weeks, as well as pericardial effusion were independent predictors of lower quality of life during the post-COVID-19 period.

## 1. Introduction

COVID-19, primarily affecting the respiratory system, caused by the severe acute respiratory syndrome coronavirus 2 (SARS-CoV-2), rapidly escalated from a localized outbreak into a global pandemic [1,2]. The disease varied from mild respiratory symptoms to severe pneumonia and acute respiratory distress syndrome (ARDS), leading to millions of deaths and significantly impacting healthcare systems, economies, and daily life worldwide [1,2]. Beyond its initial respiratory focus, the pandemic has revealed numerous health challenges, including long-term effects on various organs [3]. Notably, there is growing concern about the cardiovascular consequences of COVID-19 and their impact on quality of life in the post-pandemic era [3]. A variety of cardiac complications associated with COVID-19 have been documented, reflecting the virus’s complex impact on the cardiovascular system, including myocarditis, acute coronary syndrome, arrhythmias, and heart failure, which are classified as post-acute sequels of COVID-19 (PASCs) [4,5]. Individuals affected by COVID-19 are more vulnerable to myocardial injury, which may be due to the direct cytopathic effects of the virus or the indirect effects of circulating cytokines and other immune-mediated mechanisms on myocardium [3,6,7]. Numerous studies document prolonged non-ischemic myocardial injury and/or persistent myocarditis following apparent recuperation from the acute stage of COVID-19 [8].

Post-acute COVID-19 syndrome refers to a set of prolonged symptoms that persist in some patients four or more weeks after the initial infection or three weeks following a negative RT-PCR test for SARS-CoV-2 [9]. Recent studies categorize this syndrome into two phases: subacute COVID-19, where symptoms and abnormalities are observed 4–12 weeks after the initial infection, and chronic or post-COVID-19 syndrome, where symptoms persist beyond 12 weeks without an alternative diagnosis [9]. Common symptoms associated with post-COVID-19 syndrome include fatigue, palpitations, chest pain, weakness, sleep disturbances, dizziness, and a range of hematological disorders [10,11,12]. Around 21% of affected individuals may require rehospitalization for further treatment and evaluation [13]. This syndrome is not only a concern for those with pre-existing conditions or severe cases but also for younger, previously healthy individuals [14]. Pre-existing cardiac conditions may heighten the risk of severe complications, including potentially fatal outcomes [3]. Accurate and timely diagnosis is critical and typically relies on a combination of clinical assessments, non-invasive biomarkers, and cardiac imaging [8].

Cardiovascular magnetic resonance (CMR), as a gold standard in non-invasive cardiac diagnostics, has the ability to provide comprehensive and non-invasive insights into myocardial function and structure [8]. The ability to characterize tissue is one of the most important capabilities of CMR, as it allows for the distinction between non-ischemic and ischemic myocardial injury [15]. According to recommendations, CMR evaluation of a patients after COVID-19 infection suspected of having myocarditis should be based on established and validated criteria, such as the 2018 Lake Louise criteria [5]. The Lake Louise criteria, first published in 2009, were updated in 2018 and have since required at least one T1-based criterion (increased T1 myocardial relaxation time, extracellular volume fraction, or late gadolinium enhancement (LGE)) and at least one T2-based criterion (increased myocardial T2 relaxation time, visible myocardial edema, or increased T2 signal intensity ratio) to diagnose myocarditis on CMR [16,17]. When these criteria are met, the presence of myocarditis can be confirmed with a sensitivity of 87.5% and a specificity of 96.2% [16]. Thanks to CMR, it is possible to detect even subclinical cases, stratify risk based on prognostic factors like left ventricular ejection fraction and myocardial edema, predict outcomes, and monitor therapeutic responses [18].

In clinical practice, on a daily basis, we encounter patients who complain of persistent cardiac symptoms such as fatigue, palpitations, irregular heartbeat, and poor exercise tolerance even months after recovering from COVID-19. The inclusion of CMR in various diagnostic modalities aims to define the long-term cardiovascular consequences of infection and optimize personalized treatment strategies to improve patient quality of life. This research seeks to investigate CMR findings following COVID-19 and the impact on the quality of life of affected individuals.

## 2. Materials and Methods

### 2.1. Study Population

**Figure 1:**
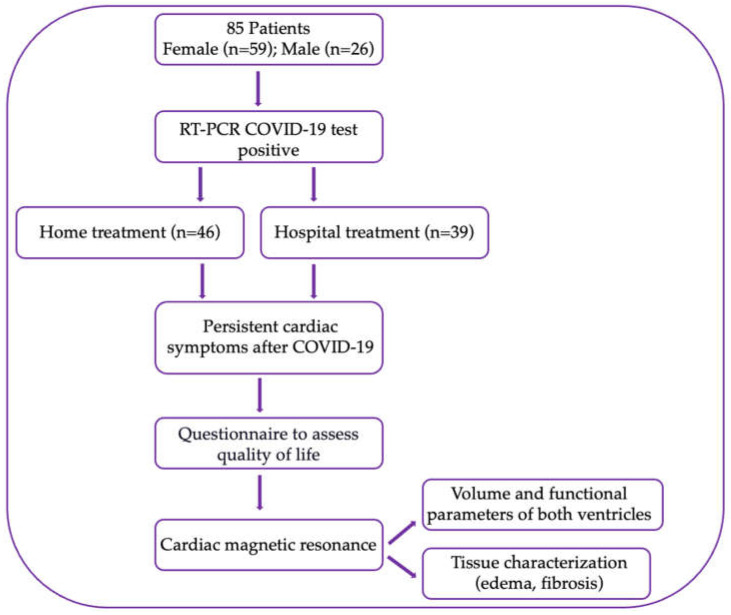
Research methodology flowchart. Abbreviations: RT-PCR—reverse transcription polymerase chain reaction test.

This study was conducted as an observational, cross-sectional study of 85 patients, with cardiovascular symptoms after severe acute respiratory syndrome coronavirus 2 infection (SARS-CoV-2), who were referred for a cardiomagnetic resonance examination (CMR), in the period from September 2020 to February 2022. This study included patients who were hospitalized for COVID-19 infection, as well as patients who did not require hospital treatment (Figure 1).

This study included consecutive patients with persistent cardiac complaints after COVID-19 infection. The most common complaints reported by patients were fatigue/exercise intolerance, palpitations, shortness of breath, chest pain, or discomfort. Due to the persistence of the aforementioned complaints, the patients were referred for cardiomagnetic resonance with suspicion of myocardial injury as part of a previous viral infection.

The inclusion criteria were as follows: (1) age ≥ 18 years; (2) previously confirmed SARS-CoV-2 infection (SARS-CoV-2 infection confirmed by reverse transcription polymerase chain reaction (RT-PCR) test within 1 month before the onset of cardiac symptoms); (3) recovery from COVID-19 infection and discharge from the hospital (patients were considered recovered if they met the following criteria: normal body temperature lasting more than 3 days, no respiratory symptoms, two consecutive negative RT-PCR test results separated by at least 24 h, and having been isolated for at least 14 days after being discharged from hospital); (4) persistent cardiac symptoms (e.g., chest pain, palpitations, dyspnea, fatigue); (5) agreement to participate and signing of informed consent.

Exclusion criteria were as follows: (1) an explanation for the patient’s complaints other than COVID-19 infection; (2) uncontrolled hypertension; (3) coronary artery disease (evidence of coronary artery stenosis >50%) or previous myocardial infarction; (4) moderate to severe valvular dysfunction; (5) previous atrial fibrillation; (6) previous heart failure; (7) contraindications for performing cardiomagnetic resonance with a contrast agent (e.g., claustrophobia; history of allergic reactions to the gadolinium-based contrast agent (GBCA); moderate, severe, or end-stage chronic kidney disease (creatinine clearance rate <30 mL/min/1.73 m^2^); non-MRI-conditional implants and devices); (8) hemodynamic instability; (9) pregnancy or breastfeeding; (10) inability to hold breath and cooperate during CMR examination.

Before the CMR examination, an interview was conducted with each patient, during which they answered questions from a dedicated questionnaire. The patients were asked about the existence of cardiovascular complaints after the COVID-19 infection, and whether they were more pronounced during the acute illness of COVID-19 or after COVID-19. To assess the quality of life during the post-COVID-19 period, a simple rating scale was used, ranging from “0” (no symptoms) to “10” (the most severe symptoms).

The present study was approved by the Ethics Committee of the University Hospital Medical Center Bezanijska Kosa (protocol code 5197/2, date of approval 21 March 2021) and informed consent was obtained from all patients. Our research was performed within the ethical guidelines of the Declaration of Helsinki of 1975 [19].

### 2.2. Cardiac MRI: Protocol and Image Analysis

All CMR examinations were performed according to a standardized protocol on a 1.5 Tesla scanner (Magnetom Avanto, Siemens Healthcare GmbH, Erlangen, Germany). The standardized protocol included sequences that allowed for functional and morphological analysis of the myocardium.

To assess the volume and functional parameters of both ventricles, cine steady-state free precession (SSFP) images were acquired in a short-axis stack (SAX), which covered the entire ventricles from base to apex. Left ventricular (LV) views included four-chamber, two-chamber, and three-chamber views, as well as a left ventricular outflow tract (LVOT). Right ventricular (RV) views included a right ventricular outflow tract (RVOT) and a two-chamber view of the right ventricle.

For tissue characterization (detection of myocardial edema and fibrosis), T2-weighted short-tau-inversion-recovery (STIR) black-blood images, myocardial mapping, and late gadolinium enhancement (LGE) images were acquired. STIR was performed using SAX stacks. Myocardial mapping (T2, native, and post-contrast T1) was performed in short-axis views. Post-contrast T1 mapping was performed approximately 15 min after contrast administration to calculate extracellular volume (ECV). An LGE image was acquired in the same planes as the cine images with a phase-sensitive inversion recovery sequence (PSIR), 8–10 min after intravenous administration of 0.2 mmol/kg gadolinium-based contrast media (GBCA).

All CMR studies were analyzed by the same two physicians (a radiologist and a cardiologist, with 7 and 5 years of experience in CMR, respectively). Any disagreements in the analysis between these two physicians were judged by senior physicians (cardiologist with more than 15 years of experience in cardiac imaging).

Post-processing of acquired CMR images and measuring of cardiac chamber volumes, mass, and function were performed using software (SyngoVia VB20A_HF05), Siemens Healthineers, Erlangen, Germany) with automated cardiac contour detection (endocardial and epicardial contour, excluding the contour of papillary muscle) along with manual correction if required. Post-processing also included assessment of myocardial edema and LGE. During the analysis of all sequences, the left ventricle was divided into 17 segments according to the American Heart Association (AHA) model of myocardial segmentation (AHA) [20].

The functional evaluation included the assessment of wall motion abnormalities, cardiac chamber volumes, mass, and function. The software automatically calculated the functional parameters of the left and right ventricles, as well as normalizing all volumes and masses according to body surface area (BSA). This resulted in ejection fraction (EF) values, indexed end-diastolic volume (EDVI), indexed end-systolic volume (ESVI), indexed values of stroke volume (SVI), cardiac output (CI), and mass.

On STIR images, focal myocardial edema was assessed as focal myocardial hyperintensity and semi-quantitatively (myocardial edema ratio (ER) was defined as the ratio between myocardial signal intensity (IS) and skeletal muscle IS, with ER > 2.0 considered abnormal). The region of interest was drawn in myocardial zones with increased signal intensity and divided by the signal intensity in skeletal muscle.

Myocardial mapping, including native T1 and T2 maps, was analyzed for myocardial global native T1 and T2 values by drawing endocardial and epicardial contours within the septal myocardium of the midventricular SAX slice. Focal native T1 and T2 values were assessed in myocardial zones with alternating signal intensity. Also, the native T1 value, along with the post-contrast T1 value, was used to calculate the extracellular volume fraction according to the following formula: ECV = (1 − hematocrit) × [ΔR1myocardium]/[ΔR1blood pool], where ΔR1_myocardium_  =  1/T1_myocardium pre-contrast_ − 1/T1_myocardium post-contrast_ and ΔR1_blood_  =  1/T1_blood pre-contrast_ − 1/T1_blood post-contrast_ [21].

The analysis of LGE images included a qualitative assessment to determine whether the LGE phenomenon was present, and if it was present, which segments it affected, and the pattern of its distribution (subepicardial, mid-wall, or transmural). In LGE-positive patients, the quantification of the LGE was also performed. This involved calculating the ratio between the LGE volume and the total volume of the LV myocardium (LGE/myocardium), expressed as a percentage of the mass of the left ventricular myocardium under the LGE.

Pericardial analysis included the detection of pericardial effusion (reported if they exceeded 5 mm in thickness) and the assessment of pericardial LGE (pericardial LGE was considered present if enhancement involved both pericardial layers, regardless of the presence of pericardial effusion).

Myocardial injury/myocarditis was diagnosed according to the 2018 Lake Louis criteria [5,16,17].

### 2.3. Statistical Analysis

Numerical data were presented as mean with standard deviation or 95% CI, or with median with percentiles. Categorical variables were summarized by absolute numbers with percentages. Differences between groups were analyzed by Students’ t test for independent samples for numerical variables with normal distribution, Mann–Whitney test for numerical variables without normal distribution, and the chi-square test for categorical variables. Univariate and multivariate linear regression analyses were used in order to determine independent predictors of lower quality of life. In all analyses, the significance level was set at 0.05. Statistical analysis was performed using IBM SPSS statistical software (SPSS for Windows, release 25.0, SPSS, Chicago, IL, USA).

## 3. Results

A total of 85 patients, mostly female (69.4%), with a mean age of 42.5 ± 13.4 years, were included in the study. Of comorbidities, 20.0% of patients had hypertension, 11.8% had diabetes mellitus, only one patient had chronic obstructive pulmonary disease (COPD), while 2.4% of patients had systemic connective tissue or inflammatory diseases. Characteristics of the study population are presented in Table 1.

Table 2 displays the patients’ cardiovascular features during the COVID-19 and post-COVID-19 periods. The median duration from the beginning of symptoms to the performance of cardiac magnetic resonance (CMR) was 120 days. The duration of cardiac symptoms exceeded 12 weeks in the majority of patients (91.8%). Out of all the patients, 35.3% received oxygen treatment during acute illness, while only 2.4% underwent mechanical ventilation. Pneumonia as a part of COVID-19 infection was observed in 55.3% of the study population. The main cardiac symptoms in the post-COVID-19 period were fatigue (81.2%), palpitations (62.4%), and irregular heartbeat (44.7%). Nine patients (10.6%) had recurrent episode of hospitalization during the post-COVID-19. The mean (95% CI) quality of life score of patients during the post-COVID-19 period was 5.2 (4.7–5.8). Additionally, in the majority of patients (57.6%), symptoms were more severe in the post-COVID-19 period than during acute illness (Table 2).

Detailed CMR findings of the study population are summarized in Table 3. At CMR, the mean ejection fraction (EF) of the left ventricle (EF LV) was 61.43 ± 5.87% and the mean ejection fraction of the right ventricle (EF RV) was 60.65 ± 7.83%. Indexed end-diastolic volume (EDVI) of the left ventricle was elevated in 23.5% of patients, indexed stroke volume (SVI) was decreased in 3.5%, and cardiac index (CI) was decreased at CMR in 1.2% of patients. None of the patients had elevated left ventricular myocardial mass.

In Table 4, detailed late gadolinium enhancement (LGE) findings are presented. The majority of patients had LGE (78.8%). Mid inferolateral (47.1%), mid anterolateral (34.1%), and basal inferolateral (25.9%) were the most prevalent myocardial segments involved by LGE in LGE-positive patients. Subepicardial and mid-wall distribution of LGE was present in 37.6% of patients with LGE, while 31.8% had isolated subepicardial distribution of LGE. Late pericardial enhancement was present in 40% of patients involved.

Pericard characteristics of the study population, as well as comprehensive assessment by multi-parametric CMR, are shown in Table 5. The mean native T1 value (diffuse) was 1020 ± 35, the mean T2 value (diffuse) was 47 ± 3, while the mean extracellular volume (ECV) fraction of the study population was 25.7 ± 1.9. Pericardial effusion was present in more than half of the patients included (51.8%).

In Table 6, CMR data are reported based on the use of oxygen therapy, mechanical ventilation, or the presence of pneumonia during acute illness. Patients who required oxygen treatment, mechanical ventilation (MV), or had pneumonia were older (*p* < 0.001), had higher body mass index (BMI) (*p* = 0.002), and more often had hypertension (*p* = 0.004) and diabetes mellitus (*p* = 0.028). Patients who did not require oxygen treatment, MV, or hadn’t pneumonia more often had basal anterior LGE on CMR (*p* = 0.007). 

Detailed CMR findings of the study population according to the presence of pericardial effusion are summarized in Table 7. Patients with pericardial effusion were younger (*p* = 0.013), more often were female (*p* = 0.002), had lower BMI (*p* = 0.001), and had lower quality of life (*p* = 0.049). The mid inferoseptal segment involved by LGE was more frequently observed in patients with pericardial effusion (*p* = 0.010). Patients with pericardial effusion had a higher mean native T1 value (diffuse) in the post-COVID-19 period (*p* = 0.029).

CMR data according to the severity of symptoms in the COVID-19/post-COVID-19 period are presented in Table 8. Patients with more severe symptoms in the post-COVID-19 period were younger (*p* = 0.022), more often were female (*p* = 0.017), had lower BMI (*p* = 0.023), and more often had lower quality of life in the post-COVID-19 period (*p* < 0.001). The basal anterior myocardial segment involved by LGE (*p* = 0.048) and pericardial effusion (*p* = 0.001) were more often observed in patients that had more severe symptoms during the post-COVID-19 period, while the apical lateral myocardial segment involved by LGE (*p* = 0.040) was more often observed in patients that had more severe symptoms during COVID-19 period.

In univariate regression analysis, female gender (*p* = 0.005), cardiovascular symptoms that last longer than 12 weeks (*p* = 0.014), presence of palpitations (*p* = 0.025), irregular heartbeat (*p* = 0.005), and unconsciousness (*p* = 0.020) were significant predictors of lower quality of life during the post-COVID-19 period. In multivariate regression analysis, predictors of lower quality of life during the post-COVID-19 period were the presence of irregular heartbeat (*p* = 0.039), cardiovascular problems that last longer than 12 weeks (*p* = 0.018), and the presence of pericardial effusion (*p* = 0.037) (Table 9).

## 4. Discussion

The following study used cardiac magnetic resonance (CMR) to evaluate the presence and spectrum of cardiac abnormalities in patients who had persistent cardiac symptoms after COVID-19 infection. Our findings revealed that most patients had abnormalities on CMR, indicative of non-ischemic myocardial injury. The predominant findings were late gadolinium enhancement (LGE) in a non-ischemic distribution and pericardial effusion, while acute myocarditis was found in a small number of patients.

Previous studies, including laboratory analyses, autopsy, and CMR findings, have shown that myocardial involvement is one of the primary manifestations of COVID-19 infection, distinguishing it from infections caused by other viruses in the coronavirus family [22,23,24,25]. Although there have been documented cases of delayed myocarditis in patients with cardiac symptoms after recovering from COVID-19, the prevalence of myocarditis in these individuals has not yet been precisely determined [1,26,27].

**Figure 2:**
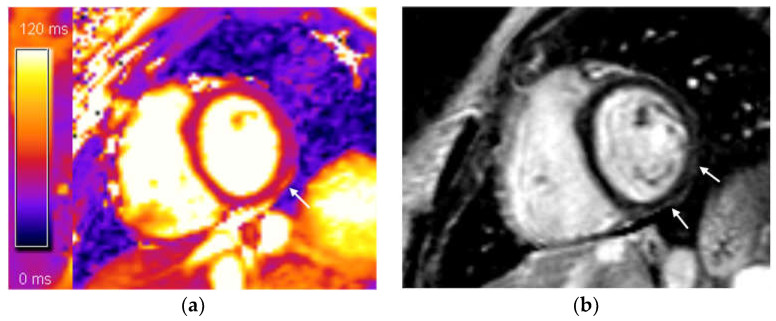
(**a**) T2 mapping, mid-ventricular short axis slice: increased focal T2 value, subepicardial distribution—a sign of subepicardial myocardial edema in the mid inferolateral segment (marked with arrow); (**b**) phase-sensitive inversion recovery (PSIR), mid-ventricular short axis slice: subepicardial (non-ischemic) distribution of late gadolinium enhancement (LGE) in mid inferior and mid inferolateral segments (marked with arrows). These CMR findings fulfilled both Lake Louise 2018 criteria for CMR diagnosis of acute myocarditis.

Our research findings suggest that acute myocarditis is relatively uncommon, as it was diagnosed in only a small number of patients. A minority (*n* = 12; 14.1%) had signs of myocardial edema, indicative of acute myocardial injury. In nearly all of these patients (*n* = 11; 12.9%), LGE was observed with a subepicardial and/or mid-wall distribution, meeting the 2018 Lake Louise criteria for diagnosing myocarditis (Figure 2) [16]. However, one patient with myocardial edema did not fully meet the criteria for myocarditis, presenting only with a T2-based marker for myocardial edema, from the modified Lake Louise criteria. This edema might be attributed to increased vascular permeability, potentially due to endothelial angiotensin-converting enzyme 2 (ACE2), which serves as a receptor for SARS-CoV-2 entry into host cells [28,29,30,31]. Additionally, some patients with myocarditis (*n* = 7; 8.23%) also presented with pericardial effusion. Both patients with confirmed myocarditis and those with myocardial edema generally had normal or mildly reduced left ventricular (LV) ejection fraction, an important prognostic marker in myocarditis [32].

In one of the earliest prospective observational cohort studies, which included 100 unselected patients who had recently recovered from COVID-19, Puntmann et al. examined the presence of myocardial injury [33]. The study revealed that 78% of the patients had abnormal CMR findings indicative of non-ischemic myocardial injury, with ongoing myocardial inflammation observed in 60% of the participants. These CMR findings were independent of pre-existing conditions, the severity and course of the acute illness, or the timing of the initial COVID-19 diagnosis. The differences between the study and our research may be attributed, at least in part, to differences in the populations being studied. In Puntmann et al., the study population included unselected individuals, with pre-existing cardiovascular conditions (diabetes mellitus, hypertension, coronary artery disease) in the majority of subjects that may have otherwise contributed to the ongoing inflammation, without significant difference in sex distribution, while the present study included patients with persistent cardiac symptoms, with cardiovascular comorbidities in the minority of subjects and with a dominant female sex distribution. Another difference is the mean duration from the onset of symptoms of COVID-19 infection until the CMR exam, i.e., in our study, it is longer and amounts to 120 days (51–209), while it is 71 days (64–92) in the study by Puntmann et al., which may be one of the factors that influenced the results. On the other hand, the results of the studies, which were conducted after the previous study, showed, similar to our results, that acute myocarditis is not a common occurrence in patients with cardiac symptoms after recovery from COVID-19 infection [1,28,34].

**Figure 3:**
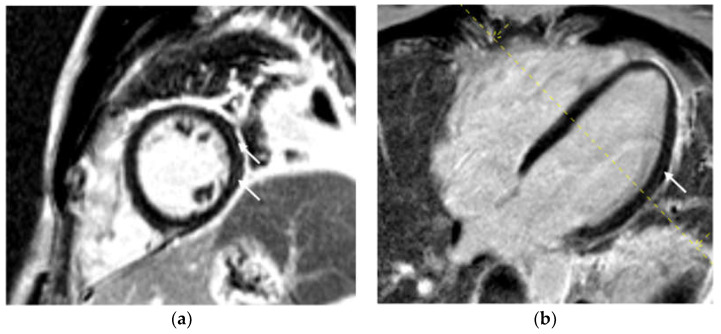
(**a**) Phase-sensitive inversion recovery (PSIR), mid-ventricular short axis slice: subepicardial and mid-wall (non-ischemic) distribution of late gadolinium enhancement (LGE) in the mid inferolateral segment (marked with arrows); (**b**) phase-sensitive inversion recovery (PSIR) long axis (four chamber): subepicardial and mid-wall (non-ischemic) distribution of late gadolinium enhancement (LGE) in the mid lateral segment (marked with arrows). This pattern of LGE indicates post-inflammatory fibrosis.

In our study, the majority of patients undergoing CMR examination had LGE in a non-ischemic distribution pattern. Specifically, LGE was predominantly localized in the inferolateral segments, particularly at the base and mid of the left ventricle (LV), with a subepicardial and/or mid-wall distribution (Figure 3). This pattern aligns with findings from studies on patients with other forms of viral myocarditis [1]. LGE is indicative of tissue inflammation, necrosis, and fibrosis, suggesting that myocarditis is a primary mechanism of direct myocardial injury caused by COVID-19 [35,36]. The simultaneous presence of LGE and edema points to active inflammation, and at this stage, LGE may not indicate definitive damage, as it often resolves upon follow-up [18,37]. Conversely, studies have shown that LGE without accompanying edema on a 6-month follow-up CMR is indicative of definitive fibrosis, representing irreversible myocardial injury [18,38,39]. Additionally, earlier studies on other types of viral myocarditis have demonstrated that LGE carries prognostic significance, often correlating with a worse prognosis [18,40].

Grani C et al., as well as Aquaro GD et al., showed an association between LGE and a worse prognosis (including sudden death, heart failure hospitalization, persistent ventricular tachycardia, implantable cardioverter/defibrillator shock, and recurrent myocarditis) in patients with myocarditis during a mean follow-up of 4.7 years, and nearly threefold increased risk of major adverse cardiovascular events (MACE) [41,42].

**Figure 4:**
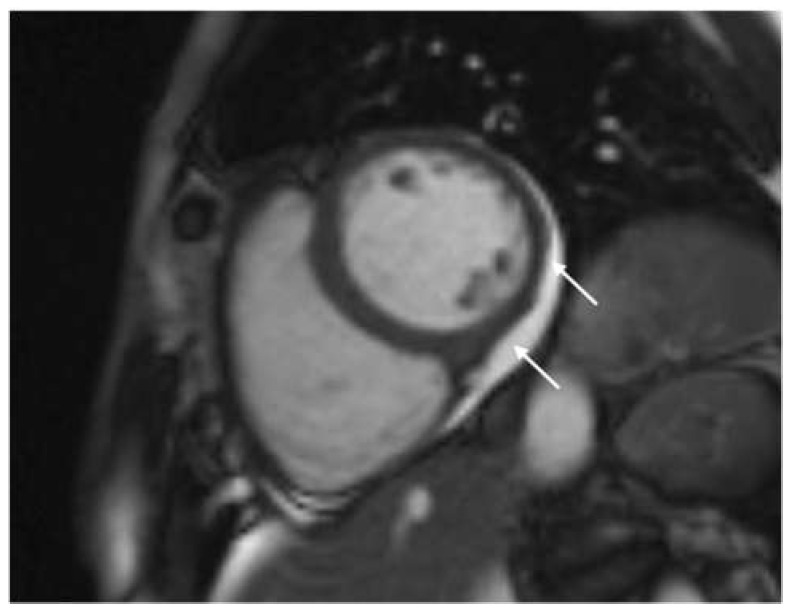
Cine sequences, mid-ventricular short axis slice: mild (<10 mm) pericardial effusion (marked with arrows).

The findings of our research showed that the phenomenon of late pericardial enhancement was present in 40% of the included patients, while mild pericardial effusion (<10 mm) was present in more than half of the included patients (51.8%) (Figure 4). These results are similar to those obtained by Brito et al. in a study that investigated the spectrum of cardiac abnormalities in previously healthy student-athletes recovering from uncomplicated COVID-19 infection [43]. The existence of pericardial effusion during COVID-19 infection is explained by several pathophysiological mechanisms: (a) by binding to the ACE2 receptor, the virus directly invades cardiomyocytes leading to myocardial injury; (b) indirectly, by means of inflammatory cytokines (tumor necrosis factor-alpha (TNF-a), interleukin (IL)-1, and IL-6) the virus leads to pericarditis or myopericarditis; (c) result of direct involvement of the pericardium; (d) consequence of acute respiratory distress syndrome (ARDS) or hypoxia [44]. Saraç et al. investigated the clinical significance of pericardial effusion (prevalence, risk factors, prognosis, late clinical outcomes, and treatment) in patients discharged after recovery from COVID-19 [44]. Their study showed that in 75.9% of patients with an initial small pericardial effusion, after a follow-up of 25 months, there was a complete resolution of the effusion, but it also showed that there was a progression of the effusion in patients with more severe disease, which consequently led to an increase in the frequency of hospitalization for recurrent cardiac and non-cardiac reasons, complications, and mortality rates. Follow-up studies after COVID-19 indicate an increased risk of cardiovascular events, including pericardial disease. It has been shown that the presence of pericardial effusion in patients in the post-COVID-19 period is one of the main risk factors and a poor prognostic indicator regarding the occurrence of complications during the acute and chronic phase [44,45].

**Figure 5:**
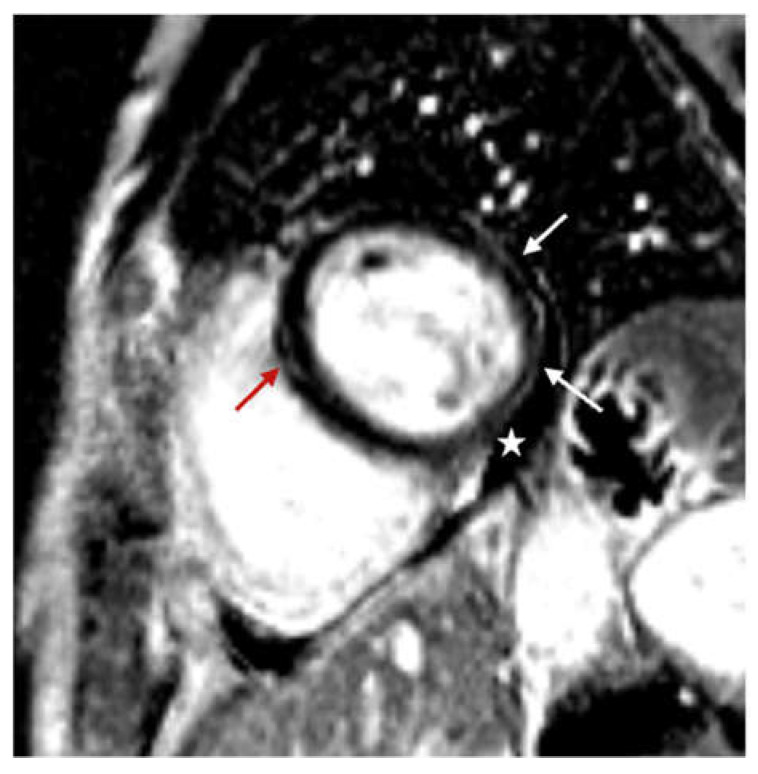
Phase-sensitive inversion recovery (PSIR), basal ventricular short axis slice: mid-wall distribution of late gadolinium enhancement (LGE) in basal anteroseptal segment (red arrow); subepicardial distribution of late gadolinium enhancement (LGE) in basal inferior and inferolateral segments (white arrow); pericardial effusion (star).

Our study compared quality of life during the post-COVID-19 period with CMR findings and showed that post-COVID-19 quality of life is worse in patients with CMR abnormalities (pericardial effusion and LGE) (Figure 5). Pericardial effusion was more often observed in patients who had more severe symptoms and lower quality of life during the post-COVID-19 period. Also, irregular heartbeat, cardiovascular symptoms that last longer than 12 weeks, as well as pericardial effusion, were independent predictors of lower quality of life during the post-COVID-19 period. It is important to note that we did not observe a significant correlation between the severity of acute COVID-19 infection and lower quality of life during the post-COVID-19 period (Table 6). Current data indicate that persistent cardiac symptoms in post-COVID-19 patients are mainly associated with the existence of active or healed myocarditis, which may be a consequence of a slower resolution of the inflammatory process or its transition to a chronic phase, which can potentially cause long-term damage to the myocardium [46,47].

The present study has several limitations: We conducted this research as a single-center study with a moderate sample size, without the existence of a control group. The research included a small number of patients with comorbidities, so the findings of our research cannot be transferred to this group. Also, this study does not provide data on the existence, type, and degree of myocardial injury during the acute phase of the disease. In addition, we did not have available data regarding the markers of myocardial injury in all patients, which prevented these data from being associated with CMR results. However, only a small number of patients had acute myocardial inflammation and minimally affected myocardium, pointing out that the usefulness of myocardial injury markers would be minimal in patients during the post-COVID-19 period.

## 5. Conclusions

In our single-center study, cardiac magnetic resonance (CMR) examination confirmed the non-ischemic cardiac injury, indicating that persistent symptoms after COVID-19 may be due to previous myocarditis. Patients with pericardial effusion had worse quality of life during the post-COVID-19 period, regardless of the severity of acute COVID-19 infection, while irregular heartbeat, cardiovascular symptoms that last longer than 12 weeks, as well as pericardial effusion, were independent predictors of lower quality of life during post-COVID-19 period. These patients may be at increased risk of heart failure or arrhythmias, so the CMR examination should be a part of the multidisciplinary diagnostic algorithm to ensure optimal and personalized treatment of patients and an optimal quality of life.

## Figures and Tables

**Table 1:** Characteristics of the study population.

Variable	(*n* = 85)
Age, mean ± sd	42.5 ± 13.4
Gender, *n* (%)	
Male	26 (30.6)
Female	59 (69.4)
BMI, mean ± sd	25.9 ± 4.3
Hypertension, *n* (%)	17 (20.0)
Diabetes mellitus, *n* (%)	10 (11.8)
COPD, *n* (%)	1 (1.2)
Systemic connective tissue or inflammatory diseases, *n* (%)	2 (2.4)

Abbreviations: BMI—body mass index; COPD—chronic obstructive pulmonary disease.

**Table 2:** Patients’ cardiovascular features during COVID-19 and post-COVID-19 periods.

Variable	
Number of days from onset of symptoms to CMR, median (interquartile range)	120 (51–209)
Number of days with cardiovascular problems	
<12 weeks	7 (8.2)
≥12 weeks	78 (91.8)
Treatment, *n* (%)	
Home treatment	46 (54.1)
Hospital treatment	39 (45.9)
Oxygen therapy, *n* (%)	30 (35.3)
Mechanic ventilation, *n* (%)	2 (2.4)
Pneumonia as a part of COVID-19 infection, *n* (%)	47 (55.3)
The main cardiac symptoms in post-COVID-19 period, *n* (%)	
Palpitations	53 (62.4)
Irregular heartbeat	38 (44.7)
Fatigue	69 (81.2)
Shortness or loss of breath	28 (32.9)
Chest pain	22 (25.9)
Fever	11 (12.9)
Dizziness and fainting	6 (7.1)
Unconsciousness	2 (2.4)
Recurrent episode of hospitalization during the period of cardiac symptoms, *n* (%)	9 (10.6)
Quality of life, mean (95% CI)	5.2 (4.7-5.8)
Severeness of symptoms, *n* (%)	
During acute infection	36 (42.4)
Post-COVID-19	49 (57.6)

Abbreviations: CMR—cardiac magnetic resonance.

**Table 3:** CMR findings.

Variable	
EF LV, mean ± sd, %	61.43 ± 5.87
EF RV, mean ± sd, %	60.65 ± 7.83
EDVI LV, *n* (%)	
Elevated	20 (23.5)
SVI LV, *n* (%)	
Decreased	3 (3.5)
CI LV, *n* (%)	
Decreased	1 (1.2)
Myocardial mass index LV in ED, *n* (%)	
Increased	0 (0.0)
Average myocardial mass index LV, *n* (%)	
Increased	0 (0.0)

Abbreviations: CMR—cardiac magnetic resonance; EF—ejection fraction; LV—left ventricle; RV—right ventricle; EDVI—indexed end-diastolic volume; SVI—indexed stroke volume; CI—cardiac index; ED—end-diastole.

**Table 4:** LGE imaging.

Variable	
LGE, *n* (%)	67 (78.8)
Myocardial segments involved by LGE, *n* (%)	
Basal anterior	5 (5.9)
Basal anteroseptal	3 (3.5)
Basal inferoseptal	6 (7.1)
Basal inferior	17 (20.0)
Basal inferolateral	22 (25.9)
Basal anterolateral	11 (12.9)
Mid anterior	2 (2.4)
Mid anteroseptal	5 (5.9)
Mid inferoseptal	10 (11.8)
Mid inferior	10 (11.8)
Mid inferolateral	40 (47.1)
Mid anterolateral	29 (34.1)
Apical anterior	1 (1.2)
Apical septal	0 (0.0)
Apical inferior	3 (3.5)
Apical lateral	3 (3.5)
Quantification of LGE (%)	
0	18 (21.7)
1	37 (44.6)
2	23 (27.7)
3	5 (6.0)
Distribution of LGE, *n* (%)	
Subepicardial	27 (31.8)
Mid-wall	7 (8.2)
Transmural	1 (1.2)
Subepicardial and mid-wall	32 (37.6)
Late pericardial enhancement, *n* (%)	34 (40.0)

Abbreviations: LGE—late gadolinium enhancement.

**Table 5:** Pericard characteristics and comprehensive assessment by multi-parametric CMR.

Variable	
Mean native T1 value (diffuse), mean ± sd	1020 ± 35
Mean T2 value (diffuse), mean ± sd	47 ± 3
Highest mean native T1 value (focal), mean ± sd	1174 ± 106
Highest mean T2 value (focal), mean ± sd	58 ± 9
ECV, mean ± sd	25.7 ± 1.9
Pericardial effusion, *n* (%)	44 (51.8)

Abbreviations: CMR—cardiac magnetic resonance; ECV—extracellular volume.

**Table 6:** Oxygen therapy, MV, or pneumonia.

Variable	Oxygen Therapy, MV, or Pneumonia		*p* Value
	No (*n* = 36)	Yes (*n* = 49)	
Age, mean ± sd	34.3 ± 10.4	48.5 ± 12.2	<0.001
Gender, *n* (%)			
Male	11 (30.6)	15 (30.6)	0.996
Female	25 (69.4)	34 (69.4)	
BMI, mean ± sd	24.3 ± 3.2	27.1 ± 4.5	0.002
Hypertension, *n* (%)	2 (5.6)	15 (30.6)	0.004
Diabetes mellitus, *n* (%)	1 (2.8)	9 (18.4)	0.028
COPD, *n* (%)	0 (0.0)	1 (2.0)	1.000
Systemic connective tissue or inflammatory diseases, *n* (%)	0 (0.0)	2 (4.1)	0.506
Quality of life, mean (95% CI)	5.5 (4.5–6.5)	5.0 (4.3–5.8)	0.437
EF LV, mean ± sd	61.7 ± 5.6	61.2 ± 6.1	0.699
EF RV, mean ± sd	60.2 ± 6.1	61.0 ± 8.9	0.646
LGE, *n* (%)	31 (86.1)	36 (73.5)	0.159
Number of segments with LGE, median (interquartile range)	2 (1–3)	2 (0–3)	0.796
Myocardial segments involved by LGE, *n* (%)			
Basal anterior	5 (13.9)	0 (0.0)	0.007
Basal anteroseptal	1 (2.8)	2 (4.1)	0.748
Basal inferoseptal	1 (2.8)	5 (10.2)	0.187
Basal inferior	7 (19.4)	10 (20.4)	0.913
Basal inferolateral	7 (19.4)	15 (30.6)	0.245
Basal anterolateral	6 (16.7)	5 (10.2)	0.380
Mid anterior	0 (0.0)	2 (4.1)	0.220
Mid anteroseptal	2 (5.6)	3 (6.1)	0.913
Mid inferoseptal	5 (13.9)	5 (10.2)	0.602
Mid inferior	5 (13.9)	5 (10.2)	0.602
Mid inferolateral	17 (47.2)	23 (46.9)	0.979
Mid anterolateral	15 (41.7)	14 (28.6)	0.208
Apical anterior	1 (2.8)	0 (0.0)	0.241
Apical inferior	0 (0.0)	3 (6.1)	0.131
Apical lateral	0 (0.0)	3 (6.1)	0.131
Quantification of LGE (%)			
≤1	24 (66.7)	31 (66.0)	0.946
>1	12 (33.3)	16 (34.0)	
Late pericardial enhancement, *n* (%)	15 (41.7)	19 (38.8)	0.788
Mean native T1 value (diffuse), mean ± sd	1015.7 ± 36.0	1022.4 ± 34.3	0.386
Mean T2 value (diffuse), mean ± sd	46.7 ± 2.8	47.6 ± 3.9	0.270
Highest mean native T1 value (focal), mean ± sd	1162.5 ± 117.4	1181.6 ± 96.4	0.412
Highest mean T2 value (focal), mean ± sd	58.6 ± 10.2	57.8 ± 7.1	0.647
ECV, mean ± sd	25.7 ± 1.9	25.6 ± 1.9	0.819
Pericardial effusion, *n* (%)	22 (61.1)	22 (44.9)	0.139

Abbreviations: MV—mechanical ventilation; BMI—body mass index; COPD —chronic obstructive pulmonary disease; EF—ejection fraction; LV—left ventricle; RV—right ventricle; LGE—late gadolinium enhancement; ECV—extracellular volume.

**Table 7:** Pericardial effusion.

Variable	Pericardial Effusion		*p* Value
	No (*n* = 41)	Yes (*n* = 44)	
Age, mean ± sd	46.2 ± 12.9	39.0 ± 13.1	0.013
Gender, *n* (%)			
Male	19 (46.3)	7 (15.9)	0.002
Female	22 (53.7)	37 (84.1)	
BMI, mean ± sd	27.5 ± 4.4	24.5 ± 3.6	0.001
Hypertension, *n* (%)	13 (31.7)	4 (9.1)	0.009
Diabetes mellitus, *n* (%)	6 (14.6)	4 (9.1)	0.428
COPD, *n* (%)	1 (2.4)	0 (0.0)	0.297
Systemic connective tissue or inflammatory diseases, *n* (%)	0 (0.0)	2 (4.5)	0.167
Quality of life, mean (95% CI)	5.8 (5.0–6.7)	4.7 (3.9–5.5)	0.049
EF LV, mean ± sd	61.7 ± 7.2	61.2 ± 4.4	0.720
EF RV, mean ± sd	58.4 ± 8.9	62.6 ± 6.1	0.019
EDVI LV, *n* (%)			
Decreased/normal values	32 (80.0)	32 (72.7)	0.434
Elevated	8 (20.0)	12 (27.3)	
SVI LV, *n* (%)			
Decreased/normal values	38 (95.0)	40 (90.9)	0.467
Elevated	2 (5.0)	4 (9.1)	
CI LV, *n* (%)			
Decreased/normal values	35 (87.5)	39 (88.6)	0.872
Elevated	5 (12.5)	5 (11.4)	
Myocardial mass index LV in ED, *n* (%)			
Decreased	22 (55.0)	23 (52.3)	0.802
Normal values	18 (45.0)	21 (47.7)	
Average myocardial mass index LV, *n* (%)			
Decreased	24 (60.0)	26 (59.1)	0.932
Normal values	16 (40.0)	18 (40.9)	
LGE, *n* (%)	31 (75.6)	36 (81.8)	0.484
Number of segments with LGE, median (interquartile range)	2 (1–3)	2 (1–3)	0.673
Myocardial segments involved by LGE, *n* (%)			
Basal anterior	2 (4.9)	3 (6.8)	0.704
Basal anteroseptal	2 (4.9)	1 (2.3)	0.515
Basal inferoseptal	3 (7.3)	3 (6.8)	0.929
Basal inferior	9 (22.0)	8 (18.2)	0.664
Basal inferolateral	14 (34.1)	8 (18.2)	0.093
Basal anterolateral	6 (14.6)	5 (11.4)	0.654
Mid anterior	2 (4.9)	0 (0.0)	0.138
Mid anteroseptal	2 (4.9)	3 (6.8)	0.704
Mid inferoseptal	1 (2.4)	9 (20.5)	0.010
Mid inferior	4 (9.8)	6 (13.6)	0.579
Mid inferolateral	16 (39.0)	24 (54.5)	0.152
Mid anterolateral	15 (36.6)	14 (31.8)	0.643
Apical anterior	0 (0.0)	1 (2.3)	0.332
Apical inferior	0 (0.0)	3 (6.8)	0.089
Apical lateral	2 (4.9)	1 (2.3)	0.515
Quantification of LGE (%)			
≤1	27 (67.5)	28 (65.1)	0.818
>1	13 (32.5)	15 (34.9)	
Late pericardial enhancement, *n* (%)	13 (31.7)	21 (47.7)	0.132
Mean native T1 value (diffuse), mean ± sd	1011 ± 30.4	1027.5 ± 37.3	0.029
Mean T2 value (diffuse), mean ± sd	47.3 ± 3.8	47.1 ± 3.2	0.836
Highest mean native T1 value (focal), mean ± sd	1186.1 ± 94.4	1161.8 ± 114.9	0.292
Highest mean T2 value (focal), mean ± sd	58.1 ± 6.6	58.2 ± 10.0	0.965
ECV, mean ± sd	25.5 ± 1.4	25.9 ± 2.2	0.399

Abbreviations: BMI—body mass index; COPD —chronic obstructive pulmonary disease; EF—ejection fraction; LV—left ventricle; RV—right ventricle; EDVI—indexed end-diastolic volume; SVI—indexed stroke volume; CI—cardiac index; ED—end-diastole; LGE—late gadolinium enhancement; ECV—extracellular volume.

**Table 8:** The severeness of symptoms in the COVID-19/post-COVID-19 period.

Variable	More Severe Symptoms		*p* Value
	COVID-19 (*n* = 36)	POST-COVID-19 (*n* = 49)	
Age, mean ± sd	46.4 ± 13.3	39.6 ± 13.0	0.022
Gender, *n* (%)			
Male	16 (44.4)	10 (20.4)	0.017
Female	20 (55.6)	39 (79.6)	
BMI, mean ± sd	27.1 ± 3.1	25.0 ± 4.8	0.023
Hypertension, *n* (%)	12 (33.3)	5 (10.2)	0.008
Diabetes mellitus, *n* (%)	6 (16.7)	4 (8.2)	0.229
COPD, *n* (%)	0 (0.0)	1 (2.0)	0.389
Systemic connective tissue or inflammatory diseases, *n* (%)	1 (2.8)	1 (2.0)	0.825
Quality of life, mean (95% CI)	6.4 (5.5–7.3)	4.4 (3.7–5.1)	<0.001
EF LV, mean ± sd	61.7 ± 4.1	61.3 ± 6.9	0.755
EF RV, mean ± sd	58.9 ± 7.2	62.0 ± 8.1	0.081
LGE, *n* (%)	30 (83.3)	37 (75.5)	0.383
Number of segments with LGE, median (interquartile range)	2 (1–3)	2 (1–3)	0.380
Myocardial segments involved by LGE, *n* (%)			
Basal anterior	0 (0.0)	5 (10.2)	0.048
Basal anteroseptal	2 (5.6)	1 (2.0)	0.386
Basal inferoseptal	3 (8.3)	3 (6.1)	0.694
Basal inferior	7 (19.4)	10 (20.4)	0.913
Basal inferolateral	12 (33.3)	10 (20.4)	0.179
Basal anterolateral	5 (13.9)	6 (12.2)	0.823
Mid anterior	1 (2.8)	1 (2.0)	0.825
Mid anteroseptal	3 (8.3)	2 (4.1)	0.410
Mid inferoseptal	2 (5.6)	8 (16.3)	0.128
Mid inferior	3 (8.3)	7 (14.3)	0.400
Mid inferolateral	19 (52.8)	21 (42.9)	0.365
Mid anterolateral	15 (41.7)	14 (28.6)	0.208
Apical anterior	0 (0.0)	1 (2.0)	0.389
Apical inferior	1 (2.8)	2 (4.1)	0.748
Apical lateral	3 (8.3)	0 (0.0)	0.040
Quantification of LGE (%)			
≤1	22 (62.9)	33 (68.8)	0.575
>1	13 (37.1)	15 (31.3)	
Late pericardial enhancement, *n* (%)	15 (41.7)	19 (38.8)	0.788
Mean native T1 value (diffuse), mean ± sd	1014.5 ± 35.7	1023.2 ± 34.3	0.261
Mean T2 value (diffuse), mean ± sd	46.5 ± 3.1	47.7 ± 3.7	0.135
Highest mean native T1 value (focal), mean ± sd	1186.1 ± 91.5	1164.2 ± 114.9	0.348
Highest mean T2 value (focal), mean ± sd	57.9 ± 6.2	58.3 ± 9.9	0.797
ECV, mean ± sd	25.3 ± 1.0	26.0 ± 1.0	0.106
Pericardial effusion, *n* (%)	11 (30.6)	33 (67.3)	0.001

Abbreviations: BMI—body mass index; COPD —chronic obstructive pulmonary disease; EF—ejection fraction; LV—left ventricle; RV—right ventricle; LGE—late gadolinium enhancement; ECV—extracellular volume.

**Table 9:** Multivariate regression analysis.

Variable	Multivariate Linear Regression Analysis		
	*β*	*t*	*p* Value
Irregular heartbeat	−0.232	−2.228	0.039
Cardiovascular problems that lasts longer than 12 weeks	−0.251	−2.408	0.018
Pericardial effusion	−0.218	−2.117	0.037

## Data Availability

The data that support the findings of this study are available from the corresponding author (M.P. or M.Z.) upon reasonable request.
